# Editorial: Metabolism and Epigenetics

**DOI:** 10.3389/fgene.2022.877538

**Published:** 2022-03-10

**Authors:** Carlos Sebastian, Joaquim S. L. Vong, Manasi K. Mayekar, Krishna S. Tummala, Indrabahadur Singh

**Affiliations:** ^1^ Department of Cell Biology, Physiology and Immunology, School of Biology, University of Barcelona, Barcelona, Spain; ^2^ Institute of Biomedicine of the University of Barcelona (IBUB), Barcelona, Spain; ^3^ School of Biomedical Sciences, The Chinese University of Hong Kong, Hong Kong SAR, China; ^4^ Oncology Discovery, AbbVie Inc., San Francisco, IL, United States; ^5^ Massachusetts General Hospital Cancer Center, Harvard Medical School, Boston, MA, , United States; ^6^ Emmy Noether Research Group Epigenetic Machineries and Cancer, Division of Chronic Inflammation and Cancer, German Cancer Research Center (DKFZ), Heidelberg, Germany

**Keywords:** chromatin, metabolites, development, disease, histone, DNA

The emerging links between cellular metabolism and epigenetics are an enthralling and timely research topic with significant implications for basic and translational research. In this editorial, we will discuss many key concepts that link a collection of review and original articles in the special issue titled ‘Metabolism and Epigenetics,’ which highlight the recent advances in our understanding of the connection between metabolism and epigenetics. We will only include a few citations, and we apologize to all our colleagues whose work we are not citing directly here. Individual review articles, to which we will refer in the text, contain references to their original work.

**FIGURE 1:**
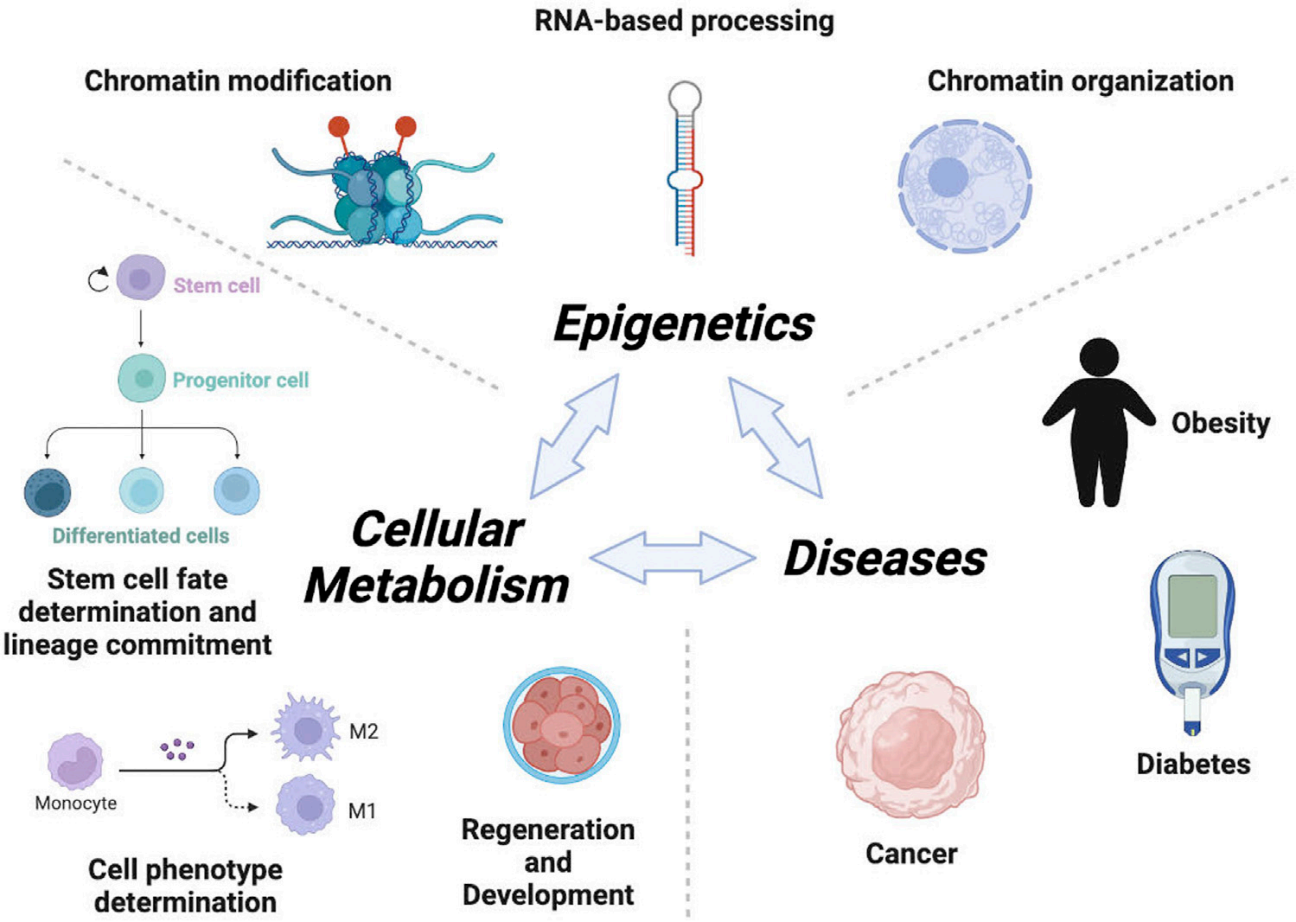
Metabolism and Epigenetics co-ordinate the development and disease. Cellular metabolism, the basic physiological unit of an organism, is subject to epigenetic regulation. Key cellular decisions such as self-renewal, stem cell fate determination, lineage commitment, and functional specification are determined by epigenetic regulation. Metabolites fuel the epigenetic machine in the form of substrates or co-factors for chromatin remodeling enzymes. Impaired epigenetic regulatory mechanisms (chromatin modification, organization and RNA-based processing, etc) in cellular metabolism may shift the physiological equilibrium into an imbalanced state, potentially contributing to disease (e.g. obesity, cancer, diabetes, etc).

During development, epigenetic mechanisms determine which genes must be turned on or off and to what extent in specific cells. In this context, functional cooperation between metabolism and epigenetics is instrumental in controlling cell fate decisions (Figure 1). It is well known that pluripotency and stem cell fate are dependent on epigenetic control of transcriptional programs involved in self-renewal and differentiation (Brunet and Rando 2017). Furthermore, cellular metabolism has been described to play an important role in controlling stemness, lineage commitment and specification (Folmes and Terzic 2016), yet how metabolism and epigenetics functionally intersect to coordinate stem cell fate is poorly understood. In this special issue, by employing a model of Müller retinal glial cells, Sanhueza-Salas et al., demonstrate that high glucose reduces SIRT6 levels leading to an increase in the expression of the pluripotent factor Sox9, which in turns promotes dedifferentiation of these cells conferring them with stem cell, proliferative and migratory properties. These results provide new clues on how metabolic inputs translate into epigenetic regulation of developmental pathways and regeneration.

Epigenetic mechanisms include DNA methylation, histone modifications, chromatin organization, and RNA-mediated processes (Figure 1). Majority of epigenetic machineries utilize specific metabolites derived from central metabolic networks as cofactors or substrates to carry out their functions (Dai et al., 2020; Morrison 2020; Huo et al., 2021; Nitsch et al., 2021). For instance, chromatin-remodeling factors such as INO80, ISWI and SWI/SNF utilize ATP for efficient restructuring of the nucleosome. Histone acetyltransferases (HATs) use acetyl co-enzyme A (acetyl-CoA) for acetylation of histones, while DNA methyltransferases (DNMTs) and lysine methyltransferases (KMTs) use S-adenosyl-L-methionine (SAM) as a co-substrate to methylate DNA and histones, respectively. The impact of metabolite levels on gene expression is highlighted by the fact that mutations in metabolic enzymes e.g. isocitrate dehydrogenase 1 and 2 (IDH1 and IDH2) result in the accumulation of onco-metabolites (2-HG) that disrupt the balance of DNA and histone methylation, resulting in widespread epigenetic dysregulation of gene expression and ultimately cancer (Wu et al., 2021). Subsequently, Luo S et al., recently demonstrated that gestational diabetes mellitus (GDM) reduced the level of SAM metabolite in murine fetal brains, thereby altering hippocampal DNA methylation and the regulation of cognition-related genes. Similarly, chromatin de-modifying enzymes such as TETs and jumonji lysine demethylases (Jmj-KDMs) require alpha-ketoglutarate (α-KG) as a co-substrate for demethylation of DNA and histones respectively. Interestingly, several metabolites derived from central metabolic networks can also have an inhibitory impact on epigenetic machineries. For instance, TETs and Jmj-KDMs are inhibited by the TCA intermediates succinate and fumarate. Moreover, Sirtuins, a family of NAD^+^-dependent protein deacylases, serve as a link between energy status and gene expression regulation by sensing NAD+/NADH levels. Thus, some epigenetic factors can act as metabolic sensors. Furthermore, changes in catalytic activity and subcellular localization of metabolic enzymes can influence epigenetic changes and gene expression programs. This emerging topic has been elegantly discussed by Ruben Boon, where the author proposes, based on recent experimental evidences, that metabolic enzymes could co-localize with chromatin factors in phase-separated nuclear domains to locally produce metabolites required for their activity. Collectively, these connections clearly demonstrate that epigenetic processes are directly dependent on many core metabolic intermediates, and thus represents an innate mechanism that links nutritional status to gene expression.

In a changing nutrient landscape, epigenetic factors regulate the activity of metabolic genes and gene products, allowing cellular metabolism to be reprogrammed (Huo, Zhang, Huang and Wang 2021; Morrison 2020; Nitsch, Zorro Shahidian and Schneider 2021). SWI/SNF epigenetic machinery activates fatty acid oxidation gene transcription during fasting/glucagon and activates lipogenic genes, promoting lipogenesis and increasing triglyceride levels in response to feeding/insulin (Zhang et al., 2016). Yu et al., recently, showed that BMI1 epigenetically promotes testosterone production in the Leydig cells by inhibiting the p38MAPK pathway. Enhancer of zeste 2 (EZH2), Histone 3 lysine 4 trimethylating (H3K27me3) epigenetic factor, was shown to affect tumor cell metabolism, including carbohydrate metabolism, amino acid metabolism and lipid metabolism (Huo, Zhang, Huang and Wang 2021). Furthermore, Liu et al., observed that the miRNA-146a rs2910164 polymorphism has been linked to atherogenic dyslipidemia. These studies clearly demonstrate that the information flow between metabolism and epigenetic processes is bidirectional.

The dysregulation of epigenetic mechanisms may cause several pathologies and contribute to metabolism-associated diseases such as obesity, diabetes, GDM, diabetic cardiomyopathy (DCM) and more (Tzika et al.; Gao et al., 2021). Deng et al., elegantly discussed that the alteration in the epigenetic machineries and/or levels of small RNAs can influence DCM pathogenesis by promoting myocardial fibrosis, cardiac electrical remodeling, metabolic reprogramming, oxidative stress and apoptosis. In line with this, Cao et al., reported that the miR-379/miR-544 cluster is involved in regulating obesity-mediated metabolic dysfunction. Furthermore, they showed that in mice, a genetic mutation in the miR-379/miR-544 cluster resulted in resistance to high-fat-diet (HFD)-induced obesity.

The articles presented in this Special Issue provide new experimental evidence on the dynamic interaction between the epigenome and the metabolome and, at the same time, put these findings into the context of emerging concepts in the field. We hope that the reader will find new provocative research hypothesis worth exploring, which will improve our understanding on the reciprocal regulation of epigenetics and metabolism in health and disease.
